# Botulism

**Published:** 1914-05

**Authors:** 


Botulism. Ch. Esmein, Paris, *Le Prog. Med.*, November 8, 1913. The term was coined about 1800 by physicians of southern Germany, to apply to poisoning by sausage and pudding (a meat pudding, not the ordinary American pudding) whence its name, by translation into Greek. The term has been used both in the narrow and in the very broad sense of any digestive disturbance due to a food but was first placed on a scientific basis by Van Ermengen, in an epidemic at Elezelles, Belgium, in 1895, when the anaerobic bacillus botulismi was isolated, the condition reproduced in animals, and the definition reduced to a specific infection. The condition is rare, mainly limited to southern Germany, the vicinity of Würtemberg, Bavaria and Baden but occasionally seen in Belgium, France and England. It occurs usually in small epidemics, involving the inhabitants of a house, garrison, boarding house, etc. (Note: The remainder of the article deals with facts theoretically well known to the profession but the preceeding well shows that the diagnosis should not be made by symptoms alone and that the term should be applied with some skepticism, and only after bacteriologic demonstration of the specific bacillus.)

